# Factors Associated with Commercial Sex Behavior among Male College Students Who Engaged in Temporary Heterosexual Behavior in Zhejiang Province, China

**DOI:** 10.1155/2022/4319194

**Published:** 2022-12-30

**Authors:** Zhongrong Yang, Weiyong Chen, Qiaoqin Ma, Xin Zhou, Wanjun Chen, Hui Wang, Meihua Jin, Tingting Jiang

**Affiliations:** ^1^Huzhou Center for Disease Control and Prevention, Huzhou, 313000 Zhejiang Province, China; ^2^Department of HIV/STD Control and Prevention, Zhejiang Provincial Center for Disease Control and Prevention, Hangzhou, 310051 Zhejiang Province, China

## Abstract

**Objective:**

This study explored the characteristics and associated factors of commercial sex behavior among male college students who engaged in temporary heterosexual behaviors in Zhejiang Province, China.

**Methods:**

The participants were male college students with temporary heterosexual behaviors. We developed an online questionnaire to collect information on demographic characteristics, sexual attitudes, sexual behaviors, and HIV/AIDS interventions through stratified cluster sampling. Chi-square (*χ*^2^) tests were performed for the different groups of participants. The occurrence of commercial sex behavior among participants was taken as the dependent variable, and logistic regression was used to analyze the factors associated with the participants' commercial sex behavior.

**Results:**

This study investigated the temporary heterosexual behavior of 424 male college students. Among them were 112 students who reported commercial sex behavior (26.42%), whose average age was 20.25 ± 1.27 years, and whose household registration of Zhejiang Province accounted for 63.39%. The results of the multivariate logistic regression analysis indicated that acceptance of commercial sex behavior (Adjusted (a) OR = 3.53, 95% CI = 1.94~6.40) and feeling at risk of contracting HIV (aOR = 6.44, 95% CI = 2.98~13.94), seeking temporary sexual partners through the Internet (aOR = 2.58, 95% CI = 1.27~5.25), consistently using condoms during sex (aOR = 0.34, 95% CI = 0.16~0.70), or using condoms sometimes/frequently during sex (aOR = 0.30, 95% CI = 0.13~0.68) were independent factors associated with male college students with temporary heterosexual behavior engaging in commercial sex behavior.

**Conclusion:**

Open sexual attitudes, seeking temporary sexual partners through the Internet, high awareness of HIV infection risk, and low condom use are associated factors for male college students engaging in commercial sex behavior. For college students' HIV/AIDS prevention and education interventions, it is necessary to strengthen the prevention of network influence, increase peer education, increase teacher participation in education, enhance college students' risk awareness, advocate for the use of condoms, and promote HIV/AIDS prevention and treatment.

## 1. Introduction

The HIV epidemic is a serious global public health problem that severely affects local social and economic development [1–3]. In recent years, the HIV epidemic among college students in China has increased rapidly, and young students have become one of the key populations for HIV/AIDS prevention and treatment. The most important transmission route for HIV infection is sexual transmission [4, 5]. As the learning environment changes, college students are transitioning from adolescence to adulthood, which increases the demand for sexual behavior, and lack of awareness of self-protection has led to a high HIV infection risk among college students [6].

In recent years, heterosexual transmission has become the primary mode of transmission for the HIV/AIDS epidemic in China. Sentinel surveillance and progress in HIV/AIDS prevention during the “13th Five-Year Plan” period in Zhejiang Province identified transmission through heterosexual commercial sex behavior and heterosexual noncommercial sex behavior as accounting for more than 50% of HIV infections. Controlling heterosexual transmission remains a difficult problem in the prevention and control of HIV/AIDS [7]. Currently, domestic college students are more tolerant of premarital sex; however, due to the lack of health education related to sexual behaviors, it may be easier for students to engage in dangerous sexual behaviors, including sexual relations with commercial sex workers and/or multiple sexual partners; however, condoms are not always used [8].

HIV/AIDS prevention for male college students is more concerned with male homosexual sex, and research on temporary heterosexual sex, especially commercial sex behavior is limited. Although a few restrictions on sex workers are in place, online and offline commercial sex transactions among male college students is rife. In view of the relationship between commercial sexual behavior and Internet dating, lack of HIV testing for both and lack of condom use that can result in HIV transmission, these behaviors pose a great risk to the physical and mental health of male college students [9, 10]. This study is the first to explore the characteristics and associated factors of commercial sex behavior among male college students who had temporary heterosexual behaviors in Zhejiang Province. This study was based on a large-scale baseline survey of 13 colleges/universities in Zhejiang Province. It provides a scientific basis for schools to formulate appropriate health education intervention strategies.

## 2. Materials and Methods

### 2.1. Study Settings

In 2018, a survey was conducted among students at 13 college students in 11 districts and cities in Zhejiang Province, including three in Hangzhou and one in each of 10 other cities. The selection of colleges and universities was recommended by the local Center for Disease Control and Prevention (CDC). A stratified cluster sampling method was used for this survey. First, three departments were selected from each university using a random number table. Subsequently, each department was divided into four levels, according to grades 1-4. At each level, 1241 classes were selected using a random number table. This study conducted a survey of 31674 college students from 13 universities in Zhejiang Province from October 2018 to November 2018. This study was reviewed and approved by the Ethics Committee of Zhejiang Provincial CDC (batch number: 2018-036). All the participants signed an informed consent form. All methods were performed in accordance with relevant guidelines and regulations.

### 2.2. Inclusion Criteria and Exclusion Criteria of the Study

The participants of this study were male college students who engaged in temporary heterosexual behaviors. Inclusion criteria were as follows: male college students whose sexual behaviors were temporarily, that of the heterosexual behavior, and who answered the questions about commercial sexual behaviors. Exclusion criteria were as follows: male college students who had temporary sexual behavior of the heterosexual sex but did not answer the question of commercial sexual behavior.

### 2.3. Study Variables and Measurements

The contents of the questionnaire developed for this study mainly included demographic and sociological characteristics, knowledge and awareness of HIV/AIDS and sexually transmitted diseases (STDs), sexual behavior, and acceptance of HIV/AIDS behavioral interventions. Questions 1-4 on HIV/AIDS and STDs' knowledge included the following: “Whether AIDS is a serious incurable disease,” “Whether the main transmission mode of HIV/AIDS among young students in China is male homosexuality,” “Whether a person is infected with HIV can be judged by appearance,” and “Whether daily life and study contact can spread HIV.” The condom use efficacy measurement has been described in our previously published article [11].

### 2.4. Data Collected

Using a cross-sectional survey, the students in the school were organized by the teachers to fill in the online electronic questionnaire. The students were sent the online link to the questionnaire and were asked to fill in the questionnaire independently according to the questionnaire headings. The survey adopted a stratified cluster sampling method. First, three departments were selected from each college/university using a random number table. Next, each department was divided into four layers according to grades 1-4. In each layer, the random number table method was used to select the classes, and 1,241 classes were selected. Before the investigation, the investigator explained the purpose, significance, investigation method, and privacy protection policy of the research, and placed this information in the opening remarks of the questionnaire. The research participants were told that the purpose of the survey was to develop strategies for students to prevent AIDS and STDs, and that survey was anonymous as well.

### 2.5. Statistical Analysis

SPSS version 21.0 software (IBM, Armonk, NY, United States) was used for the data analysis. The data of the count variables are expressed as percentage (%), and that of the measurement variables are expressed as mean ± standard deviation. The demographic characteristics of participants with or without commercial sex behavior were compared using the *χ*^2^ test. The analysis variables included demographic characteristics, sexual attitudes, acceptance of interventions, and self-efficacy about safe sex. Univariate analysis was used to analyze the factors associated with commercial sex behavior in male college students with temporary heterosexual behavior. Taking the occurrence of commercial sex behavior as the dependent variable, the variables with *P* < 0.1 and demographic characteristics from the results of univariate analysis was included as independent variables in the multivariate logistic regression analysis model. *P* < 0.05 was considered statistically significant.

## 3. Results

### 3.1. General Characteristics

Among the undergraduates surveyed, 14,320 were male students, of whom 2,734 (19.09%) undergraduates self-reported having had sex. Among male college students who had sex, 455 (16.64%) self-reported that they had temporary heterosexual sex. Among them, 424 participants affirmed their commercial sex behavior. Eventually, male college students who had temporary heterosexual behaviors and who also answered affirmatively that they had commercial sex behaviors participated in this study. There was no statistical difference in the basic characteristics between the nonincluded population and the participants included in this study.

Among the 424 male college students who had temporary sex acts with the heterosexual sex, 112 (26.42%) had experienced commercial temporary heterosexual sex. The age range of the 424 participants was 18-26 years, the average age of participants in the heterosexual commercial sex behavior group was 20.25 ± 1.27, and the average age of participants in the noncommercial sex behavior group was 20.04 ± 1.38. There were no significant differences in age, grade, hometown source, monthly living expenses, or family relationships between the two groups (*P* > 0.05, Table 1). The difference in household registration (students' origins), between other provinces and Zhejiang Province, was statistically significant (*P* < 0.05, Table 1).

### 3.2. Analysis of the Factors Associated the Commercial Behavior among Participants

In the univariate analysis (Table 2), the participants who were more likely to report commercial sex behaviors were as follows: those who had received the school's HIV testing propaganda in the last year (OR = 2.05); those who had received a self-assessment of HIV/AIDS risk conducted by the school in the past year; (OR = 1.90); those who had accepted commercial sex behavior (OR = 3.45); those who had accepted male homosexual sex (OR = 2.07); those who had wanted to know if the sexual partner was infected with HIV (OR = 1.56); those who had felt that there was a risk of contracting HIV (OR = 8.04); those who had received HIV/AIDS voluntary counseling and testing services in the past year (OR = 2.14); those who had searched for temporary sexual partners through entertainment venues (OR = 3.31) or the Internet (OR = 5.07); and those whose temporary sexual behaviors occurred after drinking (OR = 2.84). The following were more unlikely to engage in commercial sex behaviors: those who had used condoms every time (OR = 0.17) or sometimes/frequently (OR = 0.27) when having sex; those who had thought to judge whether a person was infected with HIV by appearance (OR = 0.43); and those who knew that daily life and study contact could spread HIV (OR = 0.41).

Taking the occurrence of commercial sex behavior as the dependent variable, the variables with *P* < 0.1 and demographic characteristics form the results of the univariate analysis were included as independent variables in the multivariate logistic regression analysis model. In the multivariate analysis (Table 3), the participants who were more likely to report commercial sex behaviors were as follows: those who could accept commercial sex behaviors (Adjusted (a) OR = 3.53, 95% CI = 1.94~6.40); those who had felt at risk of contracting HIV (aOR = 6.44, 95% CI = 2.98~13.94); and those who had seek temporary sexual partners through the Internet (aOR = 2.58, 95% CI = 1.27~5.25). Those who had used condoms every time (aOR = 0.34, 95% CI = 0.16~0.70), or sometimes/frequently (aOR = 0.30, 95% CI = 0.13~0.68) when having sex were less likely to engage in commercial sex behaviors.

## 4. Discussion

Our study is a cross-sectional survey of college students conducted in Zhejiang Province that reflects the characteristics and associated factors of commercial sex behavior among male college students in Zhejiang Province, China, who have temporary heterosexual behaviors. This study found that the incidence of self-reported sex among male college students in Zhejiang Province was 19.09%, the incidence of temporary sex among male college students who had heterosexual sex was 16.64% (455/2734), and the incidence of commercial sex behavior was 4.09% (112/2734). Commercial sex behavior accounted for 26.42% (112/424) of temporary sex. In recent years, studies have suggested that the incidence of self-reported male sex and commercial sex behavior has been increasing [12, 13], suggesting that with the further openness of sexual concepts, college students have a higher incidence of unsafe sex, which poses risk of contracting HIV or STDs.

Studies have demonstrated that young men with high levels of HIV/AIDS knowledge are more likely to have multiple sexual partners [14], and that unprotected sex without condoms is the most common problem among the young [15]. Furthermore, HIV high-risk sexual behaviors are inconsistent with risk perceptions [16]. The study suggested that the attitude of accepting commercial sex behavior among the commercial sex behavior group was 3.53 times that of the noncommercial sex behavior group. Furthermore, commercial sex behavior group felt their risk of contracting HIV was 6.44 times that of the noncommercial sex behavior group.

College students have sexual awareness but lack self-awareness of the importance of sexual protection [17]. Providing them with an understanding of their personal AIDS-related risk and offering the required guidance concerning their sexual risky behavior is a critical part of AIDS prevention work. However, the proportion of college students who receive sex education is not high [18]. Most are curious about participating in commercial sex. The school is an ideal place to conduct sex education for college students. It will be crucial in standardizing HIV/AIDS health education, expanding coverage and improving the effect of publicity by offering HIV/AIDS health education classes, and using the new Internet media to publicize HIV/AIDS prevention and control as well as improve knowledge and other measures [19, 20].

However, the results of this study suggest that the proportion of participants who received voluntary HIV counseling and testing was only 8.49%, suggesting that some male college students continued to have unsafe and high-risk behaviors when they had a high level of HIV/AIDS knowledge, open sexual attitudes, and high-risk awareness. Studies have shown that increasing the population's HIV/AIDS awareness and promoting the consistency of “knowledge” and “behavior” are important means to protect susceptible populations [21, 22], suggesting that consistency of knowledge, belief, and behavior should be emphasized in future HIV/AIDS health education.

The reproductive health of young students is important; it not only affects the healthy growth of students, the safety and stability of schools, and the well-being of families but also directly affects a country's future development [23]. Owing to the low capacity for safe sex among young people, the incidence of unwanted pregnancies, induced abortion, sexually transmitted diseases, and serious damage to reproductive health among young people has increased significantly [24–26]. This not only affects their physical and mental health but also brings great pressure on social security and stability.

This study found no statistical difference between the commercial sex behavior group and the noncommercial sex behavior group in terms of temporary heterosexual sex partners, but 50.5% of the commercial sex behavior group used the Internet to find temporary partners, which was 2.58 times that of the noncommercial sex behavior group. With the popularization of the Internet, some young students can easily find sexual partners and have unprotected sex [27]. This has increased challenges in HIV/AIDS prevention and control, leading to growing concerns regarding HIV transmission [28]. For Chinese students, the influence of teachers on students is higher than that of traditional media, family members, and friends; therefore, teachers' participation in school HIV/AIDS intervention education should be strengthened [29]. Additionally, we focus on establishing correct sexual ethics and sexual concepts, pay attention to how the Internet affects students, and let teachers participate fully in HIV/AIDS education intervention at school.

Consistent condom use is an effective means to prevent the spread of HIV [30, 31]; college students are aware that condom use greatly reduces the risk of HIV infection [32]. This study found that consistent condom use during temporary sex (0.30 times) and sometimes/frequently using condoms (0.34 times) reduced the occurrence of commercial sex behavior compared to those who never used condoms. However, the proportion of the commercial sex behavior group that insisted on using condoms each time during sex was only 26.8%. Studies have shown that the lack of conversion from knowledge to preventive measures in condom use among male college students is serious, and complex unsafe sexual behaviors increase the difficulty of intervention [33–35]. Therefore, to educate college students on HIV/AIDS prevention, it is necessary to emphasize that the individual is the first person responsible for their own health. Promoting use of condoms is essential to preventing them from being infected by such diseases.

This study had several limitations. First, this research is a cross-sectional survey, and causal inferences cannot be made regarding the associated factors. In addition, the contents of this survey were self-reports of the research participants; there might have been some phenomena, such as recall bias, which might have affected the interpretation of the results. Finally, the questionnaire design of this study was not specifically aimed at male college students with temporary behaviors regarding the heterosexual sex. Simultaneously, the questionnaire lacked a variable relating to the time of the first sexual behavior, which may have had an impact on the results of the research. Therefore, the results of this study need to be verified using a large sample and multicenter survey research.

## 5. Conclusions

Male college students who engaged in commercial sex behavior had a high degree of openness in their sexual attitudes, high awareness of HIV infection risk, and high proportion of success in finding temporary partners through the Internet. However, they reported low condom use during sex and low HIV testing rates. It is recommended to develop and implement health education programs based on the characteristics of the population, strengthen the prevention of network influence, increase the intensity of peer education, increase teachers' participation in health literacy education, enhance the risk awareness of college students, and promote the consistency of prevention regarding “knowledge” and “behavior” to reduce the physical and mental impact of STDs in students.

## Figures and Tables

**Table 1:** Demographic characteristics of participants.

Variables	Commercial sex behavior group (*n* = 112)		Noncommercial sex behavior group (*n* = 312)		*x* ^2^	*P*
	*n*	%	*n*	%		
Age (years)	20.25 ± 1.27		20.04 ± 1.38			
18-19	30	26.8	111	35.6	2.87	0.09
20-26	82	73.2	201	64.4		
Grades					2.837	0.418
Freshman	18	16.1	73	23.4		
Sophomore	40	35.7	106	34.0		
Junior	41	36.6	104	33.3		
Senior	13	11.6	29	9.3		
Household registration					3.987	0.046
Other provinces	41	36.6	83	26.6		
Zhejiang Province	71	63.4	229	73.4		
Hometown					1.738	0.187
Rural area	58	51.8	184	59.0		
Town/city	54	48.2	128	41.0		
Monthly living expenses (RMB)					2.916	0.233
≦1000	37	33.0	89	28.5		
1001-1500	26	23.2	99	31.7		
≧1501	49	43.8	124	39.7		
Family relationship						
Harmonious	90	80.4	246	78.8	0.114	0.735
General/disharmonious/divorced	22	19.6	66	21.2		

^∗^RMB: renminbi.

**Table 2:** Univariate analysis of commercial sex behavior among 424 participants.

Variables	Commercial sex behavior group (*n* = 112)		Noncommercial sex behavior group (*n* = 312)		Univariate analysis	
	*n*	%	*n*	%	OR(95% CI)	*P*
Whether AIDS is a serious incurable disease?						
No	26	23.2	67	21.5	Ref.	
Yes	86	76.8	245	78.5	0.91 (0.54-1.51)	0.703
Whether the main transmission mode of HIV/AIDS among young students in China is male homosexuality?						
No	58	51.8	174	55.8	Ref.	
Yes	54	48.2	138	44.2	1.17 (0.76-1.81)	0.468
Whether a person is infected with HIV can be judged by appearance?						
No	36	32.1	53	17.0	Ref.	
Yes	76	67.9	259	83.0	0.43 (0.26-0.71)	0.001
Whether daily life and study contact can spread HIV?						
No	35	31.3	49	15.7	Ref.	
Yes	77	68.8	263	84.3	0.41 (0.25-0.68)	0.001
Have you learned about HIV/AIDS through the school's campus internet network in the past year?						
No	84	75.0	235	75.3	Ref.	
Yes	28	25.0	77	24.7	1.02 (0.62-1.68)	0.946
Have you received the school's HIV testing propaganda in the last year?						
No	23	20.5	108	34.6	Ref.	
Yes	89	79.5	204	65.4	2.05 (1.23-3.43)	0.006
Have you received an HIV/AIDS risk self-assessment conducted by the school in the past year?						
No	44	39.3	172	55.1	Ref.	
Yes	68	60.7	140	44.9	1.90 (1.22-2.95)	0.004
Could you accept one-night stand?						
No	23	20.5	70	22.4	Ref.	
Yes	89	79.5	242	77.6	1.12 (0.66-1.90)	0.677
Could you accept commercial sex behavior?						
No	28	25.0	167	53.5	Ref.	
Yes	84	75.0	145	46.5	3.45 (2.13-5.60)	<0.001
Could you accept male-to-male sexual behavior?						
No	93	83.0	284	91.0	Ref.	
Yes	19	17.0	28	9.0	2.07 (1.11-3.88)	0.021
Could you want to know whether the sexual partner is infected with HIV^∗^						
No	45	40.5	160	51.6	Ref.	
Yes	66	59.5	150	48.4	1.56 (1.01-2.43)	0.046
Could you feel there was a risk of contracting HIV^∗^						
No	78	69.6	295	94.9	Ref.	
Yes	34	30.4	16	5.1	8.04 (4.22-15.31)	<0.001
Condom use self-efficacy measurement^∗^						
No confidence	21	18.8	75	24.1	Ref.	
Have confidence	37	33.0	109	35.0	1.21 (0.66-2.23)	0.537
Very confident	54	48.2	127	40.8	1.52 (0.85-2.71)	0.157
Whether to accept HIV/AIDS voluntary counseling and testing?						
No	97	86.6	291	93.3	Ref.	
Yes	15	13.4	21	6.7	2.14 (1.06-4.32)	0.033
Could you know that the CDC provides HIV testing services?						
No	19	17.0	35	11.2	Ref.	
Yes	93	83.0	277	88.8	0.62 (0.34-1.13)	0.12
Temporary partner type^∗^						
Student	71	64.5	203	66.3	Ref.	
Student + social worker	22	20.0	64	20.9	0.98 (0.56-1.71)	0.951
Social worker	17	15.5	39	12.7	1.25 (0.66-2.34)	0.494
How to find temporary sexual partners^∗^						
Nonnetwork (except entertainment venues)	18	16.2	137	44.8	Ref.	
Internet	56	50.5	84	27.5	5.07 (2.80-9.21)	<0.001
Entertainment venues	37	33.3	85	27.8	3.31 (1.77-6.19)	<0.001
Have you ever engaged in temporary sex after drinking^∗^						
No	39	35.5	187	60.9	Ref.	
Yes	71	64.5	120	39.1	2.84 (1.80-4.46)	<0.001
Whether had condom use during sex^∗^						
Never use	36	32.1	29	9.4	Ref.	
Sometimes/frequently used	46	41.1	140	45.5	0.27 (0.15-0.48)	<0.001
Always use	30	26.8	139	45.1	0.17 (0.09-0.33)	<0.001
Could you discuss condom use during sex?^∗^						
No	26	23.2	87	28.3	Ref.	
Yes	86	76.8	220	71.7	1.31 (0.79-2.17)	0.296
Have sex with a fixed partner in the past year^∗^						
No	39	35.1	99	31.8	Ref.	
Yes	72	64.9	212	68.2	0.86 (0.55-1.36)	0.525

^∗^There is missing data.

**Table 3:** Multivariate analysis of commercial sex behavior among 424 participants.

Variables	Commercial sex behavior group (*n* = 112)		Noncommercial sex behavior group (*n* = 312)		Univariate analysis		Multivariate analysis	
	*n*	%	*n*	%	OR(95% CI)	*P*	OR(95% CI)	*P*
Age (years)								
18-19	30	26.8	111	35.6	Ref.		Ref.	
20-26	82	73.2	201	64.4	1.51 (0.94-2.44)	0.091	1.42 (0.79-2.56)	0.241
Hometown								
Rural area	58	51.8	184	59.0	Ref.		Ref.	
Town/city	54	48.2	128	41.0	1.34 (0.87-2.07)	0.188	1.37 (0.80-2.33)	0.249
Household registration								
Other provinces	41	36.6	83	26.6	Ref.		Ref.	
Zhejiang Province	71	63.4	229	73.4	0.63 (0.40-0.99)	0.047	0.57 (0.32-1.01)	0.051
Whether a person is infected with HIV can be judged by appearance?								
No	36	32.1	53	17.0	Ref.		Ref.	
Yes	76	67.9	259	83.0	0.43 (0.26-0.71)	0.001	0.79 (0.37-1.68)	0.536
Whether daily life and study contact can spread HIV?								
No	35	31.3	49	15.7	Ref.		Ref.	
Yes	77	68.8	263	84.3	0.41 (0.25-0.68)	0.001	0.71 (0.31-1.59)	0.398
Have you received the school's HIV testing propaganda in the last year?								
No	23	20.5	108	34.6	Ref.		Ref.	
Yes	89	79.5	204	65.4	2.05 (1.23-3.43)	0.006	1.69 (0.88-3.25)	0.116
Have you received an HIV/AIDS risk self-assessment conducted by the school in the past year?								
No	44	39.3	172	55.1	Ref.		Ref.	
Yes	68	60.7	140	44.9	1.90 (1.22-2.95)	0.004	1.06 (0.58-1.94)	0.839
Could you accept commercial sex behavior?								
No	28	25.0	167	53.5	Ref.		Ref.	
Yes	84	75.0	145	46.5	3.45 (2.13-5.60)	<0.001	3.53 (1.94-6.40)	<0.001
Could you accept male-to-male sexual behavior?								
No	93	83.0	284	91	Ref.		Ref.	
Yes	19	17.0	28	9	2.07 (1.11-3.88)	0.021	0.51 (0.21-1.24)	0.136
Could you want to know whether the sexual partner is infected with HIV^∗^								
No	45	40.5	160	51.6	Ref.		Ref.	
Yes	66	59.5	150	48.4	1.56 (1.01-2.43)	0.046	1.07 (0.62-1.84)	0.817
Could you feel there was a risk of contracting HIV^∗^								
No	78	69.6	295	94.9	Ref.		Ref.	
Yes	34	30.4	16	5.1	8.04 (4.22-15.31)	<0.001	6.44 (2.98-13.94)	<0.001
Whether to accept HIV/AIDS voluntary counseling and testing?								
No	97	86.6	291	93.3	Ref.		Ref.	
Yes	15	13.4	21	6.7	2.14 (1.06-4.32)	0.033	1.75 (0.69-4.43)	0.24
How to seek temporary sexual partners^∗^								
Nonnetwork (except entertainment venues)	18	16.2	137	44.8	Ref.		Ref.	
Internet	56	50.5	84	27.5	5.07 (2.80-9.21)	<0.001	2.58 (1.27-5.25)	0.009
Entertainment venues	37	33.3	85	27.8	3.31 (1.77-6.19)	<0.001	1.92 (0.92-4.01)	0.084
Have you ever engaged in temporary sex after drinking^∗^								
No	39	35.5	187	60.9	Ref.		Ref.	
Yes	71	64.5	120	39.1	2.84 (1.80-4.46)	<0.001	1.63 (0.91-2.91)	0.098
Whether had condom use during sex^∗^								
Never use	36	32.1	29	9.4	Ref.		Ref.	
Sometimes/frequently used	46	41.1	140	45.5	0.27 (0.15-0.48)	<0.001	0.34 (0.16-0.70)	0.004
Always use	30	26.8	139	45.1	0.17 (0.09-0.33)	<0.001	0.30 (0.13-0.68)	0.004

^∗^There is missing data.

## Data Availability

The analyzed data used to support the findings of this study are available from the corresponding author upon request.

## Abbreviations

HIV: Human immunodeficiency virus
AIDS: Acquired immune deficiency syndrome
STDs: Sexually transmitted diseases
RMB: Renminbi
CDC: Center for Disease Control and Prevention.
